# Perioperative entecavir for patients with HBV-related hepatocellular carcinoma and low levels of viral DNA: analysis using propensity score matching

**DOI:** 10.18632/oncotarget.15395

**Published:** 2017-02-16

**Authors:** Bao-Hong Yuan, Ru-Hong Li, Wei-Ping Yuan, Bang-De Xiang, Ming-Hua Zheng, Tian Yang, Jian-Hong Zhong, Le-Qun Li

**Affiliations:** ^1^ Department of General Surgery, Yan’An Hospital Affiliated to Kunming Medical University, Kunming, China; ^2^ Department of Hepatobiliary Surgery, Affiliated Tumor Hospital of Guangxi Medical University, Nanning, China; ^3^ Department of Hepatology, Liver Research Center, The First Affiliated Hospital of Wenzhou Medical University, Wenzhou, China; ^4^ Department of Hepatobiliary Surgery, Eastern Hepatobiliary Surgery Hospital, Second Military Medical University, Shanghai, China

**Keywords:** hepatocellular carcinoma, Hepatitis B virus, entecavir, HBV reactivation, liver function

## Abstract

The safety and efficacy of perioperative antiviral therapy for patients with hepatitis B virus related hepatocellular carcinoma and low serum levels of hepatitis B virus DNA are unknown. This retrospective study compared serum levels of hepatitis B virus DNA, liver function, morbidity, and length of hospital stay between patients who underwent hepatic resection alone and patients who received entecavir therapy before and after resection (*n* = 44 in each group). Propensity score matching was used to reduce confounding due to baseline differences between the groups. Hepatitis B virus reactivation during follow-up, which lasted a median of 6.1 months, occurred in one patient in the entecavir group (2.3%) and 11 patients in the resection-only group (25%; *P* = 0.02). Liver function, especially alanine aminotransferase levels, recovered much faster in the entecavir group. This group also showed a slightly lower rate of morbidity (*P* = 0.081) as well as significantly shorter overall hospital stay (20.1 ± 4.9 vs 24.9 ± 13.2 days; *P* = 0.028) and postoperative hospital stay (11.4 ± 1.9 vs 16.8 ± 13.1 days; *P* = 0.008). These results from this pilot study suggest that patients with hepatitis B virus related hepatocellular carcinoma and low levels of hepatitis B virus DNA are at risk of hepatitis B virus reactivation following resection, and that perioperative entecavir therapy can safely and effectively reduce this risk. Such therapy also appears to improve liver function and shorten hospitalization.

## INTRODUCTION

Hepatitis B virus (HBV) infection is a global health challenge. In addition to being a serious infection, it is a major risk factor for death from cirrhosis and hepatocellular carcinoma (HCC) [1–2]. Pathogenesis of HBV-related liver disease depends on the dynamic relationship between the degree of viral replication and the host immune response to HBV [3]. In most HBV-infected carriers, the virus remains in an inactive state [4], but HBV reactivation often occurs in inactive HBsAg carriers during immunosuppressive therapy against malignancies [5]. HBV reactivation has even been reported among patients with HBsAg-negative (occult) infection, following anticancer chemotherapy [6]. In fact, any immunosuppressive treatment can provoke HBV reactivation, giving rise to serological profiles typical of acute hepatitis [7]. This risk of reactivation likely reflects that low-level viral replication persists for a long time in liver cells and peripheral blood mononuclear cells [8–9]. Reducing the risk of HBV reactivation is important because reactivation can lead to severe and even fulminant clinical prognosis [10].

HBV infection is one of the main risk factors for HCC. Studies have reported that patients with HBV-related HCC and high serum levels of HBV DNA can suffer HBV reactivation as a result of HCC treatments [11–15]. Administration of antiviral therapy to such patients before surgery or chemotherapy can reduce the risk of reactivation [16–17]. Whether the same is true for patients with HBV-related HCC and low levels of HBV DNA is unclear [18–19], and current official guidelines do not recommend antiviral therapy for such patients [20–23].

This retrospective study aimed to investigate whether perioperative entecavir antiviral therapy could reduce the risk of HBV reactivation after hepatic resection in patients with HBV-related HCC and low serum levels of viral DNA. We also examined whether such therapy could improve recovery of liver function, reduce postoperative morbidity, and shorten length of hospital stay. In comparing patients who underwent hepatic resection with or without perioperative entecavir therapy, we analyzed propensity score-matched patient pairs in order to reduce confounding from baseline differences.

## RESULTS

### Study population

During the enrollment period, 218 patients met the inclusion and exclusion criteria, including 44 who received entecavir therapy. The remaining 174 patients who did not receive entecavir therapy were assigned to the control group. The two groups were not comparable along some baseline demographic and clinical characteristics. Therefore 1:1 propensity score matching was used to generate 44 pairs of patients similar across all characteristics examined (all *P* > 0.05; Table 1).

**Table 1:** Perioperative baseline characteristics

Variable	Entecavir group	Control group	*P*
Gender, M/F	32/12	38/6	0.113
Median age, yr	49	49	1.000
HBsAg, ng/ml	328 ± 125	350 ± 133	0.437
HbeAg, +/-	6/38	4/40	0.502
AFP, +/-	32/12	32/12	1.000
HBV DNA, copies/mL	7315 ± 1106	5615 ± 3251	0.555
Urea nitrogen, mmol/L	5.3 ± 1.5	5.2 ± 1.1	0.732
Creatinine, μmol/L	56.7 ± 43.8	78.8 ± 26.8	0.066
Prothrombin time, s	13.35 ± 0.98	14.7 ± 0.99	0.385
Leukocyte count, x10^9^/L	5.95 ± 2.10	6.34 ± 1.86	0.355
Platelet count, x10^9^/L	194.7 ± 99.2	206.9 ± 104.8	0.578
Total bilirubin, μmol/L	12.2 ± 5.5	9.9 ± 4.0	0.131
Direct bilirubin, μmol/L	5.36 ± 3.15	4.88 ± 2.06	0.389
Albumin, g/L	43.2 ± 7.4	42.5 ± 4.1	0.726
Alanine transaminase, U/L	32.7 ± 16.8	43.3 ± 20.1	0.067
Glutamic transaminase, U/L	41.2 ± 18.4	53.5 ± 31.6	0.070
Blood loss, ml	509.1 ± 205.5	436 ± 293.8	0.614
Largest tumor size, cm	7.0 ± 3.3	7.4 ± 3.2	0.579
Single tumor, n (%)	38 (86)	36 (82)	0.560
First hepatic portal occlusion, present/absent	34/10	34/10	1.000
Tumor capsule, present/absent	26/18	30/14	0.375
Portal vein tumor thrombus, present/absent	14/30	10/34	0.161
Percutaneous ethanol injection, present/absent	6/38	10/34	0.269
Perioperative blood transfusion, present/absent	16/28	12/32	0.360
Cirrhosis, severe/moderate/mild/none	2/12/24/6	0/18/22/4	0.297
Ascites, present/absent	2/42	4/40	0.398
Surgical procedure, anatomic/non-anatomic	24/20	24/20	1.000
Hepatectomy type, major/minor	16/28	16/28	1.000
BCLC stage, 0/A/B/C	0/26/4/14	0/26/4/14	0.741

AFP, alpha-fetoprotein; BCLC, Barcelona Clinical Liver Cancer; HBV, hepatitis B virus

### Outcomes

Follow-up lasted a median of 6.1 months (range, 3.1 to 12.8). During that time, no obvious entecavir-related side effects were observed. HBV reactivation occurred in a significantly larger proportion of control patients [11/44, (25%)] than entecavir patients [1/44 (2.3%), *P* = 0.02]. Of the 15 reactivation cases, 8 occurred in the first week after hepatic resection. Patients who experienced HBV reactivation received entecavir therapy.

Before resection, patients in the two groups had similar serum levels of HBV DNA (*P* = 0.561). On days 4 and 8 after resection, DNA levels were significantly lower in the entecavir group than in the control group (all *P* < 0.05; Table 2). In fact, DNA levels in the control group rose to 18.4×10^4^ copies/mL on day 8.

**Table 2:** Serum levels of HBV DNA (copies/mL) before and after hepatic resection

Time point	Entecavir group	Control group	*P*
3 days before resection	7.3×10^3^ ± 1.1×10^3^	5.6×10^3^ ± 3.3×10^3^	0.561
4 days after resection	0.8×10^3^ ± 0.5×10^3^	18.2×10^3^ ± 16.5×10^3^	0.012
8 days after resection	0.6×10^3^ ± 0.5×10^3^	18.4×10^3^ ± 13.1×10^3^	0.005
30 days after resection	< 500	11.7×10^3^ ± 9.3×10^3^	< 0.001

Before resection, patients in the two groups had similar normal liver function, but by day 8 after resection, entecavir patients had a significantly lower level of alanine aminotransferase (*P* = 0.012) and shorter prothrombin time. On days 4, 8 and 30, the two groups had similar total bilirubin and albumin levels (all *P* > 0.05; Table 3).

**Table 3:** Liver function parameters after hepatic resection

Parameter	Entecavir group	Control group	*P*
Alanine aminotransferase, U/L			
4 days after resection	149.2 ± 104.2	276.2 ± 198.2	0.066
8 days after resection	53.7 ± 27.8	90.3 ± 56.5	0.012
30 days after resection	31.8 ± 22.4	43.7 ± 19.2	0.421
Total bilirubin, μmol/L			
4 days after resection	23.1 ± 20.3	19.5 ± 10.6	0.497
8 days after resection	15.1 ± 14.0	18.0 ± 14.6	0.503
30 days after resection	13.7 ± 8.2	16.1 ± 6.8	0.620
Albumin, g/L			
4 days after resection	36.8 ± 22.5	32.5 ± 3.0	0.368
8 days after resection	34.0 ± 4.0	32.6 ± 2.9	0.180
30 days after resection	37.0 ± 8.1	36.1 ± 6.7	0.314
Prothrombin time, s			
4 days after resection	14.0 ± 1.4	14.9 ± 1.6	0.055
8 days after resection	13.7 ± 1.6	14.4 ± 1.4	0.164
30 days after resection	12.4 ± 2.8	13.4 ± 9.2	0.427

No liver failure or other severe complications occurred in the entecavir group, whereas two control patients (4.5%) suffered liver failure and another four (9.1%) developed hepatorenal syndrome due to liver dysfunction. Overall, entecavir patients had slightly lower postoperative morbidity than control patients (*P* = 0.081).

Entecavir patients showed significantly shorter overall hospital stay, as well as shorter postoperative hospital stay, than control patients (*P* = 0.028; Table 4).

**Table 4:** Length of hospital stay and complications after resection

Parameter	Entecavir group	Control group	*P*
Patients experiencing postoperative complications, n (%)	12 (27.3)	20 (45.5)	0.080
Overall hospitalization, days	20.09 ± 4.94	24.86 ± 13.24	0.028
Postoperative hospitalization, days	11.41 ± 1.87	16.77 ± 13.06	0.008

## DISCUSSION

HBV reactivation can degrade liver function, aggravate cirrhosis, increase the incidence and severity of potentially life-threatening complications, and increase the risk of HBV-related HCC recurrence [24–25]. For these reasons, both Asian and Western official guidelines [20–23, 26] recommend simultaneous comprehensive antitumor and antiviral treatment for HCC patients with high levels of HBV DNA. Whether perioperative entecavir therapy is also safe and effective for patients with low serum levels of HBV DNA is unknown. In this small retrospective study involving propensity score-matched patients, we found entecavir therapy to be safe and effective at reducing occurrence of HBV reactivation, improving liver function recovery, reducing postoperative morbidity and shortening hospital stay.

The most widely-used definition of HBV reactivation is an abrupt rise in HBV DNA levels by at least 1 log_10_ from baseline [27]. The rate of this reactivation was significantly higher in our control group than our entecavir group. While the specific mechanism of HBV reactivation is unknown, it appears to be associated with immunosuppression [26, 28]. Since hepatic resection involves greater immunosuppression than some other HCC treatments, such as radiofrequency ablation, resection is associated with relatively high risk of HBV reactivation [29–31]. The ability of perioperative entecavir therapy to reduce risk of postoperative HBV reactivation correlates with our finding that HBV DNA levels abruptly declined in the entecavir group - with levels undetectable in half of patients — whereas they increased in control patients, all of whom showed detectable levels.

Patients with HBV reactivation are thought to recover liver function more slowly than patients who do not suffer reactivation [32]. For example, previous work reported that by day 30 after hepatic resection, the proportion of HCC patients achieving a predefined level of liver function recovery was significantly higher among those who did not suffer HBV reactivation than among those who did [33, 34]. While our small sample size precluded us from comparing liver function recovery between patients with or without HBV reactivation, we did find that entecavir patients had significantly lower levels of alanine aminotransferase than control patients on day 8 after resection, implying faster recovery of liver function. Serum alanine aminotransferase is released into the blood as a result of liver cell necrosis caused when HBV DNA replication causes an acute inflammatory response. Even though levels of alanine aminotransferase rose in entecavir patients immediately after resection, they were not so as high as in the control group, and they recovered faster than in the control group. At the same time, the two groups of patients had similar levels of total bilirubin and albumin as well as similar prothrombin time at days 4 and 8 after resection. These results likely reflect the relatively short follow-up time or the weak correlation with entecavir therapy.

The rate of postoperative morbidity was 18.2% lower in the entecavir group than in the control group, and most of the excess cases in the control group showed poor liver function recovery. In fact, 4.5% of control patients experienced liver failure and 9.1% experienced hepatorenal syndrome due to liver dysfunction. These results suggest that perioperative entecavir therapy can improve recovery of liver function, thereby reducing the rate of postoperative morbidity, especially liver dysfunction and liver failure. These findings are consistent with previous studies [35–37]. The faster recovery of liver function and lower rate of postoperative morbidity in the entecavir group help explain their shorter overall hospitalization, as well as shorter postoperative hospitalization. Postoperative hospitalization can reflect recovery more accurately than overall hospitalization, since preoperative procedures and examinations can vary among patients.

The findings of our study should be interpreted with caution because of the relatively short follow-up of approximately 6 months. Longer follow-up is needed to analyze the effects of perioperative entecavir therapy on liver function in greater detail, as well as measure possible effects of such therapy on recurrence rate and overall survival. Previous study found that postoperative antiviral therapy with lamivudine or entecavir can improve long-term overall survival of patients with HBV-related HCC [38–40]. Further study is also needed to verify whether our findings of the clinical benefit of antiviral therapy apply also to patients with HBV-related HCC and low levels of HBV DNA after transarterial chemoembolization, radiofrequency ablation, or liver transplantation.

In conclusion, our retrospective study provides evidence that perioperative entecavir therapy can accelerate recovery of liver function, reduce HBV reactivation and morbidity as well as shorten hospital stay after hepatic resection of patients with HBV-related HCC and low serum levels of HBV DNA.

## PATIENTS AND METHODS

### Patients

Consecutive patients with HBV-related HCC who underwent hepatic resection between October 2014 and December 2015 at the Yan’An Hospital Affiliated to Kunming Medical University or the Affiliated Tumor Hospital of Guangxi Medical University were considered for inclusion in the study. Patients were included in the present study if they satisfied all the following inclusion criteria: (a) they underwent hepatic resection for HCC; (b) HCC was confirmed by histopathology; (c) they had preserved liver function; (d) they were positive for serum HBsAg and HBV DNA levels exceeded 500 copies/mL (100 IU/mL) without reaching the antiviral therapy standard of the Asian-Pacific Clinical Practice Guidelines on the Management of Hepatitis B [21]; and (e) they received either perioperative entecavir therapy or no antiviral therapy. Patients were excluded if they met any of the following criteria: (a) they had received chemotherapy or any other antitumor treatments prior to hepatic resection; (b) they had previously received antiviral therapy with interferon or nucleos(t)ide analogue within 12 months before resection; (c) they suffered surgical infection after resection; or (d) they had autoimmune disease, malignant tumors in other organs, or other severe disease.

This study was approved by the Ethics Committee of both participating hospitals, and it complied with the Declaration of Helsinki.

### Definitions and surgical procedure

Liver function was compared before and after hepatectomy based on prothrombin time and based on levels of alanine aminotransferase, total bilirubin, and albumin. HBV reactivation was defined as an alanine aminotransferase level > 2 Upper Limit of Normal in combination with either an abrupt rise in HBV replication of 1 log_10_ from baseline or an absolute value > 20 000 IU/mL [27]. Levels of HBV DNA in serum were quantified using real-time PCR coupled to a fluorescence assay in a commercial hepatitis detection kit (DaAn Gene, Guangzhou, China). The manufacturer-specified lower limit of quantification was 500 copies/mL [41].

Hepatic resection was performed as described [42–44]. Indications for blood transfusions [45, 46] and criteria for hospital discharge [47] were as described.

### Patient allocation and perioperative management

In this study period, patients who satisfied the inclusion criteria of this study were treated with entecavir therapy unless the patient requested without it. Actually, most patients had unwilling to receive antiviral therapy before resection because of their poor economic condition. Patients in the entecavir group received entecavir (0.5 mg/d; Zhengda Tianqing, Lianyungang, China) starting 3 days before hepatic resection and for at least 1 month afterwards. Patients in the control group did not receive any antiviral therapy. Control patients who experienced HBV reactivation were considered for entecavir therapy [26]. None of the patients in our study received immunological therapy during the perioperative period.

### Data collection

At 3 days before resection, all patients were subjected to the following tests: HBsAg and its antibody, hepatitis B e antigen and its antibody, antibody to HBV core antigen, HBV Pre-S1, HBV DNA, liver function (alanine aminotransferase, albumin, total bilirubin, prothrombin time), routine blood analysis, renal function, coagulation function, and alpha-fetoprotein. These analyses were repeated at days 4, 8 and 30 after resection. Follow-up was continued at least 3 months after hospital discharge.

Primary outcomes included the occurrence of HBV reactivation, liver function recovery, and postoperative morbidity. The secondary outcome was length of hospital stay.

### Statistical analysis

Continuous data were reported as mean and standard deviation and compared between groups using the independent-samples *t* test. Categorical data were reported as percentages and compared between groups using the chi-squared or Fisher's exact tests as appropriate. The threshold of significance was defined as two-tailed *P* < 0.05. Data were analyzed using SPSS 20.0 (IBM, USA).

In order to reduce bias in patient selection, propensity analysis was carried out using logistic regression to create propensity scores for patients with and without entecavir therapy in an observational database [48]. Logistic regression was applied to clinical variables differing significantly between patients with and without entecavir therapy, and propensity scores were generated along a continuous range from 0 to 1. The model was then used to provide a one-to-one nearest-neighbor match between patients with and without entecavir therapy [49, 50]. All variables in Table 1 were included in propensity score matching.

## References

[1] Ott JJ, Stevens GA, Groeger J, Wiersma ST Global epidemiology of hepatitis B virus infection: new estimates of age-specific HBsAg seroprevalence and endemicity. Vaccine.

[2] Yang JD, Roberts LR Hepatocellular carcinoma: A global view. Nat Rev Gastroenterol Hepatol.

[3] Mori S, Fujiyama S Hepatitis B virus reactivation associated with antirheumatic therapy: Risk and prophylaxis recommendations. World J Gastroenterol.

[4] Yim HJ, Lok AS Natural history of chronic hepatitis B virus infection: what we knew in 1981 and what we know in 2005. Hepatology.

[5] Shouval D, Shibolet O Immunosuppression and HBV reactivation. Semin Liver Dis.

[6] Yeo W, Chan TC, Leung NW, Lam WY, Mo FK, Chu MT, Chan HL, Hui EP, Lei KI, Mok TS, Chan PK Hepatitis B virus reactivation in lymphoma patients with prior resolved hepatitis B undergoing anticancer therapy with or without rituximab. J Clin Oncol.

[7] Raimondo G, Pollicino T, Cacciola I, Squadrito G Occult hepatitis B virus infection. J Hepatol.

[8] Cabrerizo M, Bartolome J, Caramelo C, Barril G, Carreno V Molecular analysis of hepatitis B virus DNA in serum and peripheral blood mononuclear cells from hepatitis B surface antigen-negative cases. Hepatology.

[9] Rehermann B, Ferrari C, Pasquinelli C, Chisari FV The hepatitis B virus persists for decades after patients’ recovery from acute viral hepatitis despite active maintenance of a cytotoxic T-lymphocyte response. Nat Med.

[10] Umemura T, Tanaka E, Kiyosawa K, Kumada H Mortality secondary to fulminant hepatic failure in patients with prior resolution of hepatitis B virus infection in Japan. Clin Infect Dis.

[11] Zhong JH Nucleos(t)ide analogue therapy for HBV-related HCC after hepatic resection: clinical benefits and unanswered questions. Tumour Biol.

[12] Dan JQ, Zhang YJ, Huang JT, Chen MS, Gao HJ, Peng ZW, Xu L, Lau WY Hepatitis B virus reactivation after radiofrequency ablation or hepatic resection for HBV-related small hepatocellular carcinoma: a retrospective study. Eur J Surg Onc.

[13] Huang G, Lai EC, Lau WY, Zhou WP, Shen F, Pan ZY, Fu SY, Wu MC Posthepatectomy HBV reactivation in hepatitis B-related hepatocellular carcinoma influences postoperative survival in patients with preoperative low HBV-DNA levels. Ann Surg.

[14] Kim BK, Park JY, Kim DY, Kim JK, Kim KS, Choi JS, Moon BS, Han KH, Chon CY, Moon YM, Ahn SH Persistent hepatitis B viral replication affects recurrence of hepatocellular carcinoma after curative resection. Liver Int.

[15] Huang W, Zhang W, Fan M, Lu Y, Zhang J, Li H, Li B Risk factors for hepatitis B virus reactivation after conformal radiotherapy in patients with hepatocellular carcinoma. Cancer Sci.

[16] Loomba R, Rowley A, Wesley R, Liang TJ, Hoofnagle JH, Pucino F, Csako G Systematic review: the effect of preventive lamivudine on hepatitis B reactivation during chemotherapy. Ann Intern Med.

[17] Zhong JH, Ma L, Li LQ (2015). Postoperative Antiviral Therapy With Nucleos(t)ide Analogs in Patients With Hepatitis B Virus-related Hepatocellular Carcinoma. Ann Surg.

[18] Zhong JH, Yang T, Xiang BD, Li LQ, Ma L Antiviral therapy for hepatitis B virus-related hepatocellular carcinoma after surgery: A comment for moving forward. World J Hepatol.

[19] Ke Y, Wang L, Li LQ, Zhong JH Nucleos(t)ide analogues to treat hepatitis B virus-related hepatocellular carcinoma after radical resection. World J Hepatol.

[20] European Association For The Study Of The Liver EASL clinical practice guidelines: Management of chronic hepatitis B virus infection. J Hepatol.

[21] Liaw YF, Kao JH, Piratvisuth T, Chan HL, Chien RN, Liu CJ, Gane E, Locarnini S, Lim SG, Han KH, Amarapurkar D, Cooksley G, Jafri W Asian-Pacific consensus statement on the management of chronic hepatitis B: a 2012 update. Hepatol Int.

[22] Lok AS, McMahon BJ Chronic hepatitis B: update 2009. Hepatology.

[23] Perrillo RP, Gish R, Falck-Ytter YT American Gastroenterological Association Institute technical review on prevention and treatment of hepatitis B virus reactivation during immunosuppressive drug therapy. Gastroenterology.

[24] Chan SL, Wong VW, Qin S, Chan HL Infection and Cancer: The Case of Hepatitis B. J Clin Oncol.

[25] Chen G, Lin W, Shen F, Iloeje UH, London WT, Evans AA Past HBV viral load as predictor of mortality and morbidity from HCC and chronic liver disease in a prospective study. Am J Gastroenterol.

[26] Sarin SK, Kumar M, Lau GK, Abbas Z, Chan HL, Chen CJ, Chen DS, Chen HL, Chen PJ, Chien RN, Dokmeci AK, Gane E, Hou JL Asian-Pacific clinical practice guidelines on the management of hepatitis B: a 2015 update. Hepatol Int.

[27] Liaw YF, Chu CM Hepatitis B virus infection. Lancet.

[28] Xie ZB, Zhu SL, Peng YC, Chen J, Wang XB, Ma L, Bai T, Xiang BD, Li LQ, Zhong JH Postoperative hepatitis B virus reactivation and surgery-induced immunosuppression in patients with hepatitis B-related hepatocellular carcinoma. J Surg Oncol.

[29] Weng DS, Zhou J, Zhou QM, Zhao M, Wang QJ, Huang LX, Li YQ, Chen SP, Wu PH, Xia JC Minimally invasive treatment combined with cytokine-induced killer cells therapy lower the short-term recurrence rates of hepatocellular carcinomas. J Immunother.

[30] Ng KK, Lam CM, Poon RT, Shek TW, To JY, Wo YH, Ho DW, Fan ST Comparison of systemic responses of radiofrequency ablation, cryotherapy, and surgical resection in a porcine liver model. Ann Surg Oncol.

[31] Liu F, Dan J, Zhang Y, Chen M, Huang J, Xie R Hepatitis B reactivation after treatment for HBV-related hepatocellular carcinoma: comparative analysis of radiofrequency ablation versus hepatic resection. [Article in Chinese]. Zhonghua Gan Zang Bing Za Zhi.

[32] Huang L, Li J, Yan J, Sun J, Zhang X, Wu M, Yan Y Antiviral therapy decreases viral reactivation in patients with hepatitis B virus-related hepatocellular carcinoma undergoing hepatectomy: a randomized controlled trial. J Viral Hepat.

[33] Huang L, Li J, Lau WY, Yan J, Zhou F, Liu C, Zhang X, Shen J, Wu M, Yan Y Perioperative reactivation of hepatitis B virus replication in patients undergoing partial hepatectomy for hepatocellular carcinoma. J Gastroenterol Hepatol.

[34] Gong WF, Zhong JH, Lu SD, Wang XB, Zhang QM, Ma L, Zhang ZM, Xiang BD, Li LQ (2017). Effects of antiviral therapy on post-hepatectomy HBV reactivation and liver function in HBV DNA-negative patients with HBV-related hepatocellular carcinoma. Oncotarget.

[35] Nakamoto S, Kanda T, Nakaseko C, Sakaida E, Ohwada C, Takeuchi M, Takeda Y, Mimura N, Iseki T, Wu S, Arai M, Imazeki F, Saito K Reactivation of hepatitis B virus in hematopoietic stem cell transplant recipients in Japan: efficacy of nucleos(t)ide analogues for prevention and treatment. Int J Mol Sci.

[36] Yuen MF Anti-viral therapy in hepatitis B virus reactivation with acute-on-chronic liver failure. Hepatol Int.

[37] Hwang JP, Barbo AG, Perrillo RP Hepatitis B reactivation during cancer chemotherapy: an international survey of the membership of the American Association for the Study of Liver Diseases. J Viral Hepat.

[38] Ke Y, Ma L, You XM, Huang SX, Liang YR, Xiang BD, Li LQ, Zhong JH Antiviral therapy for hepatitis B virus-related hepatocellular carcinoma after radical hepatectomy. Cancer Biol Med.

[39] Zhong JH, Zhong QL, Li LQ, Li H Adjuvant and chemopreventive therapies for resectable hepatocellular carcinoma: a literature review. Tumour Biol.

[40] Huang G, Lau WY, Wang ZG, Pan ZY, Yuan SX, Shen F, Zhou WP, Wu MC Antiviral therapy improves postoperative survival in patients with hepatocellular carcinoma: a randomized controlled trial. Ann Surg.

[41] Xie ZB, Wang XB, Fu DL, Zhong JH, Yang XW, Li LQ Postoperative hepatitis B virus reactivation in hepatitis B virus-related hepatocellular carcinoma patients with hepatitis B virus DNA levels < 500 copies/mL. Onco Targets Ther.

[42] Zhong JH, Li H, Xiao N, Ye XP, Ke Y, Wang YY, Ma L, Chen J, You XM, Zhang ZY, Lu SD, Li LQ Hepatic resection is safe and effective for patients with hepatocellular carcinoma and portal hypertension. PLoS One.

[43] Zhong JH, Xiang BD, Gong WF, Ke Y, Mo QG, Ma L, Liu X, Li LQ Comparison of long-term survival of patients with BCLC stage B hepatocellular carcinoma after liver resection or transarterial chemoembolization. PLoS One.

[44] Zhu SL, Ke Y, Peng YC, Ma L, Li H, Li LQ, Zhong JH Comparison of long-term survival of patients with solitary large hepatocellular carcinoma of BCLC stage A after liver resection or transarterial chemoembolization: a propensity score analysis. PLoS One.

[45] Yang T, Lu JH, Lau WY, Zhang TY, Zhang H, Shen YN, Alshebeeb K, Wu MC, Schwartz M, Shen F Perioperative blood transfusion does not influence recurrence-free and overall survivals after curative resection for hepatocellular carcinoma: A Propensity Score Matching Analysis. J Hepatol.

[46] Zhong JH, Xiang BD, Li LQ Blood transfusion and postoperative complications: a cautionary comment. Transl Gastroenterol Hepatol.

[47] You XM, Mo XS, Ma L, Zhong JH, Qin HG, Lu Z, Xiang BD, Wu FX, Zhao XH, Tang J, Pang YH, Chen J, Li LQ Randomized Clinical Trial Comparing Efficacy of Simo Decoction and Acupuncture or Chewing Gum Alone on Postoperative Ileus in Patients With Hepatocellular Carcinoma After Hepatectomy. Medicine (Baltimore).

[48] Austin PC An Introduction to Propensity Score Methods for Reducing the Effects of Confounding in Observational Studies. Multivariate Behav Res.

[49] Zhong JH, Ke Y, Gong WF, Xiang BD, Ma L, Ye XP, Peng T, Xie GS, Li LQ Hepatic resection associated with good survival for selected patients with intermediate and advanced-stage hepatocellular carcinoma. Ann Surg.

[50] Mirici-Cappa F, Gramenzi A, Santi V, Zambruni A, Di Micoli A, Frigerio M, Maraldi F, Di Nolfo MA, Del Poggio P, Benvegnù L, Rapaccini G, Farinati F, Zoli M Treatments for hepatocellular carcinoma in elderly patients are as effective as in younger patients: a 20-year multicentre experience. Gut.

